# Formation Generation for Multiple Unmanned Vehicles Using Multi-Agent Hybrid Social Cognitive Optimization Based on the Internet of Things

**DOI:** 10.3390/s19071600

**Published:** 2019-04-02

**Authors:** Zheng Yao, Sentang Wu, Yongming Wen

**Affiliations:** 1 School of Automation Science and Electrical Engineering, Beihang University, Beijing 100191, China; woost@buaa.edu.cn; 2 Science and Technology on Information Systems Engineering Laboratory, Beijing Institute of Control & Electronics Technology, Beijing 100038, China; wenyongming_buaa@foxmail.com

**Keywords:** Internet of Things, formation generation, distributed information fusion, autonomous collaboration, social cognitive optimization, multi-agent system

## Abstract

Multi-agent hybrid social cognitive optimization (MAHSCO) based on the Internet of Things (IoT) is suggested to solve the problem of the generation of formations of unmanned vehicles. Through the analysis of the unmanned vehicle formation problem, formation principles, formation scale, unmanned vehicle formation safety distance, and formation evaluation indicators are taken into consideration. The application of the IoT enables the optimization of distributed computing. To ensure the reliability of the formation algorithm, the convergence of MAHSCO has been proved. Finally, computer simulation and actual unmanned aerial vehicle (UAV) formation generation flight generating four typical formations are carried out. The result of the actual UAV formation generation flight is consistent with the simulation experiment, and the algorithm performs well. The MAHSCO algorithm based on the IoT is proved to be able to generate formations that meet the mission requirements quickly and accurately.

## 1. Introduction

With the continuous maturation of intelligence equipment technology, the deepening of swarm intelligence research, and the improvement of distributed multi-sensor fusion technology, more and more unmanned operations are beginning to adopt the collaborative self-organization of multi-vehicle clusters. The collaborative work of multiple unmanned vehicles requires effective decision-making and management. Some groups of unmanned vehicles can be controlled by humans. However, the situation for most groups of unmanned vehicles is that the nature of tasks, communication ability, decision-making time, etc., make it impossible for human beings to participate in decision making. These groups of unmanned vehicles must therefore realize unmanned decision-making and management. Generally, each member of such groups has certain decision-making ability through devices such as embedded processors, however how the whole group makes collaborative decisions is still a difficult problem.

The problem of the generation of formations of multiple unmanned vehicles is a typical group decision-making problem with distributed information fusion. However, the formation generation problem is less studied than popular problems such as target allocation, formation control, and sensor networks. Reference [1] analyzed the formation structure of multiple UAVs in different numbers and different models. Study [2] established an adjustable unmanned vehicle formation model according to the environment. Study [3] analyzed the different characteristics of different unmanned vehicle formations by observing and studying birds. Reference [4] proposed an unmanned vehicle formation structure that could adapt to large-scale intelligent vehicles. Study [5] used taxonomy to give a collaborative evaluation method for a UAV group in an unknown confrontation environment. Reference [6] described an unmanned vehicle formation construction method without communication through relative navigation. Study [7] described a task-based unmanned vehicle formation construction method of leader-follower. Study [8] presented a novel approach to a position control problem in rigid formations of nonholonomic UAVs. Reference [9] described a track guidance method for formation flight of UAVs. Study [10] presented a multirotor aircraft formation flight control method with collision avoidance capability. Reference [11] presented a coordinated flight control method for hypersonic glide vehicles with multiple no-fly-zone constraints. Existing researches on formations of unmanned vehicles have paid more attention to formation maintenance and have achieved rich results. Study [12] proposed a scheme for sharing sensor information among multiple UAVs. Study [13] proposed a new multi-UAV task allocation method. Reference [14] designed a network based on multiple UAVs. The above studies conducted an in-depth analysis of the formation of unmanned vehicles. The contents analyzed include: the type of formation, the formation evaluation, the method of the UAVs to reach the formation, the communication method used by the formation, etc. However, formation in many studies is manually specified or determined according to the motion relationship of the formation unmanned vehicles. Little attention was paid to the formation generation methods and algorithms. This paper will supplement studies for the unmanned vehicles formation research in this part.

In essence, the formation generation problem is a decision-making problem. For the problem of unmanned group decision-making, there are two kinds of general schemes: centralized decision-making and distributed decision-making.

In centralized decision-making groups, a member usually acts as the "decision center". The “decision center” determines the actions of all members of the group. This approach is simple and easy to implement, but it has two fatal shortcomings: (1) the huge calculations in the “decision center”; and (2) the group’s requirement of high communication ability requirement. For example, even the members farthest from the “decision center” must communicate directly with the “decision center”. This leads to a high demand for the effective distance of communication equipment. To overcome the above two shortcomings, some researchers have adopted the method of "distributed computing and centralized decision-making", while others have used a method of information communicating step by step among the groups. However, these methods bring difficulties: the former reduced the calculation amount of the "decision center" and increased the communication traffic, and the latter increased the communication delay.

The fundamental way to overcome the shortcomings of centralized decision-making is distributed decision-making. Distributed decision-making means that every member of a group calculates and makes decisions. Each member only communicates with members within a certain range. On this basis, members act according to certain established rules. With the action of the above process, the group finally achieves a state of good coordination among members and achieves the task requirements together.

The emerging Internet of Things technology provides an effective way to organize UAVs. Study [15] introduced the basic method of UAV-based IoT taking crowd surveillance as an example. Reference [16] introduced how to select the appropriate UAVs for a particular IoT task. Study [17] proposed algorithms for UAV path planning using IoT sensor networks. Study [18] explained the network architecture for the UAV-based IoT. The above studies proved the feasibility of UAVs group and IoT collaborative application from the adaptation of IoT and UAV, network structure and UAV applications under IoT support. Based on these studies, it is an effective method to apply the distributed decision algorithm of the UAVs group in IoT.

When the group has both centralized decision making and distributed decision making capabilities, a mixed strategy can be used. The mixed strategy has a variety of specific solutions, such as: when a member’s computing performance is high and the communication state is good, the group adopts centralized decision, and when the communication quality is degraded, the distributed decision is adopted. Even groups can allocate decision tasks to members based on events trigger. Mixed strategy is an advanced decision-making form based on a highly intelligent group.

The collaboration of unmanned vehicle groups can be divided into two types: collaboration with formation and collaboration without formation. In a task requiring the collaborative completion of an unmanned vehicle group, unmanned vehicles are often randomly placed in a certain area or start moving toward a concentration area from a certain location at the beginning of the task. For the collaboration problem with formation, whether the unmanned vehicles can gather from the initial position and generate the appropriate formation is the prerequisite for the successful implementation of the task. This paper aims to study this problem.

A typical specific problem can be described as follows: A certain number of unmanned vehicles are randomly located in a certain area. To accomplish a task which requires a variety of functions, they need to adopt an appropriate formation and move to the task area in this formation. Therefore, it is necessary for each member to move into the appropriate formation through distributed decision-making. The Internet of Things (IoT) makes this kind of distributed decision possible. In the IoT, each member can be calculated as part of an algorithm to achieve fast distributed operations.

It should be noted that, in this paper, unmanned vehicles include UAVs, other unmanned vehicles, and other robots with similar characteristics. Most unmanned vehicles move in two dimensions, although others, such as UAVs, move in three dimensions. When the formation needs to be adjusted, three-dimensional moving objects have more room for adjustment. For example, when there is a colliding danger in the formation, members of the two-dimensional formation have to avoid collision by velocity adjustment. However, the members in the three-dimensional formation can easily resolve the crisis by adjusting the height. Moreover, for many tasks, the efficiency of two-dimensional formation is higher than that of three-dimensional formations, such as searching for the ground, spraying plants, and so on. Therefore, this paper focuses on the 2D formation problem. And the formation problem in three-dimensional space can be decomposed into the two-dimensional formation problems of multiple horizontal planes at different levels.

## 2. Problem Analysis

### 2.1. Formation Principle

In a previous study [19], we proposed that, although the collaborative formation of different groups has its own characteristics, the following three basic principles are generally followed:

(1) The need for collaboration formation: the overall effectiveness of multiple unmanned vehicles is significantly improved compared to that of multiple unmanned vehicles that perform missions independently or without collaboration;
(2) The maximization for overall effectiveness: There is an optimal balance between the individual effectiveness of an unmanned vehicle and the overall effectiveness of the formation. Additionally, there is also an optimal balance between the overall effectiveness and the total cost. The goal of collaborative formation is to maximize the overall effectiveness of the formation;
(3) The integrity of the formation: When unmanned vehicles perform tasks in collaborative formation, it is necessary to take into account the difference in performance of unmanned vehicles. In the process of information acquisition and sharing, decision-making, and collaborative action, all unmanned vehicles in formation should be taken into account.

These principles above are important guidelines for formation generation.

### 2.2. Formation System and Formation Generation Function

**Figure 1:**
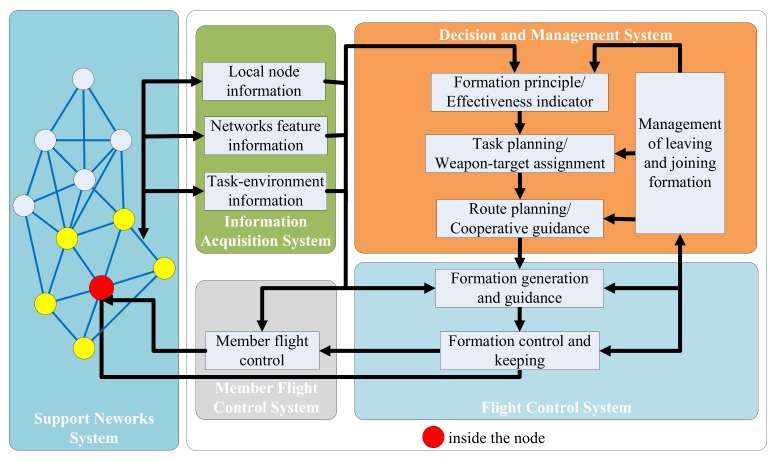
Structure of the unmanned vehicle collaboration formation system.

Previous studies have shown that the complete unmanned vehicle collaboration formation system (the structure is shown in Figure 1) should include five parts:

• Information Acquisition System (IAS);
• Decision and Management System (DMS);
• Flight Control System (FCS);
• Member Flight Control System (MFCS);
• Support Networks System (SNS);

It can be seen that the unmanned vehicle collaboration formation system has the characteristics of a small IoT. Additionally, its decision-making process also depends on the support of the IoT.

**Figure 2:**
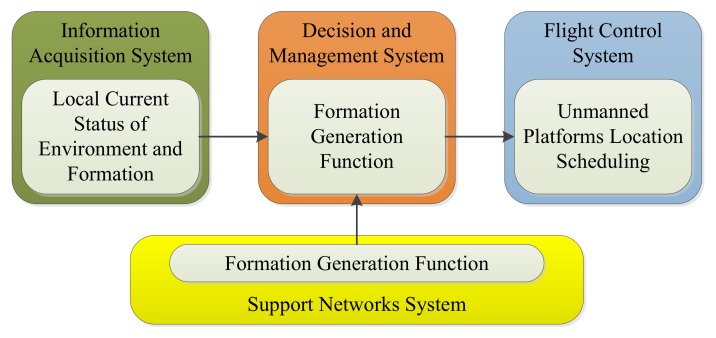
Input and output of the formation generation function.

The formation generation function should be completed by DMS. The input and output of this function are shown in Figure 2.

As shown in Figure 2, IAS should provide enough information about task requirements, formation members, formation states, and other necessary messages, while FCS should control the movement of formation members according to the generated formation pattern to form a designed formation.

### 2.3. Formation Size and Foundation Formation

Formation size has a great influence on the generation of the formation. In theory, as long as IAS, SNS, and other systems can provide enough support, and the external environment allows, any number of unmanned vehicles can form a formation that meets certain requirements. However, if there are too many formation members, it is still necessary to divide the whole formation into small groups to improve the control efficiency.

**Figure 3:**
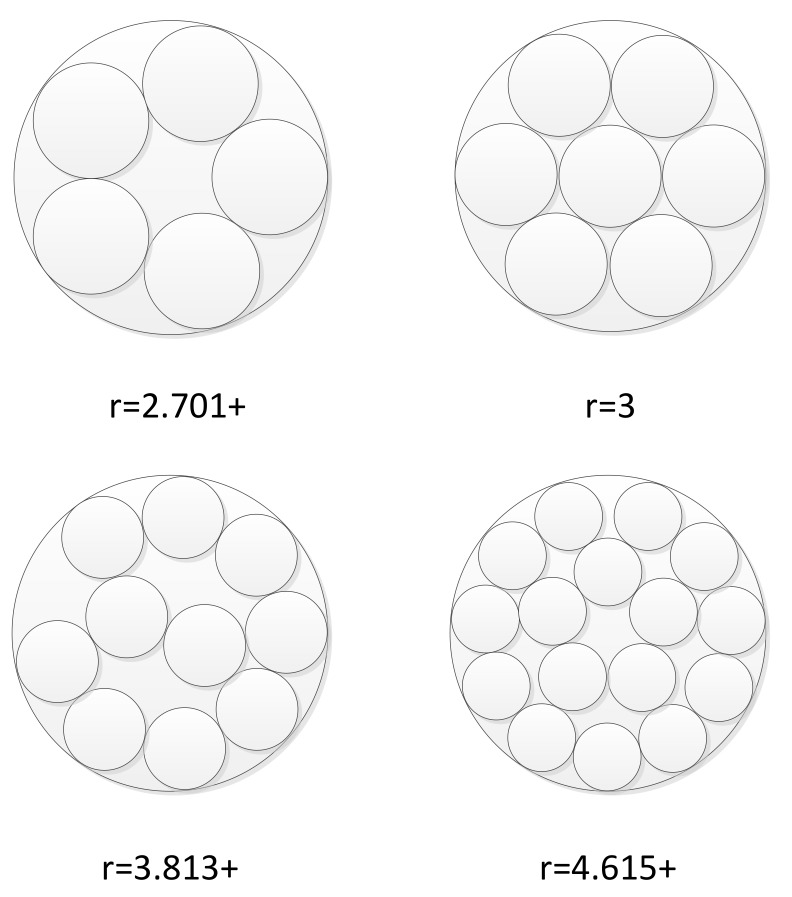
Some of the solutions to the “small circle in a large circle problem”. The term r is the ratio of the diameter of the large circle to that of the small circle.

Although different types of unmanned vehicles have different dynamic characteristics, it can be approximately considered that the maneuver space required by each unmanned vehicle in formation is a circle centered on the unmanned vehicle. The classic “circles packing in a circle” problem involves determining the maximum radius of the small circle when a certain number of small circles are placed in a unit circle. Some of the solutions to this problem are shown in Figure 3.

When this problem is applied to the formation of unmanned vehicles, the problem becomes: given the radius of the small circle, how many small circles can be placed within the unit circle? Study [20] has shown that the average number of adjacent circles around a small circle at the center is between 6 and 8. In other words, it is appropriate to divide the large-scale formation into small groups containing about 6–8 unmanned vehicles.

### 2.4. Algorithm Requirements

According to the above analysis, the formation generation algorithm should meet the following requirements:

Faster computing speed: Unmanned vehicles such as unmanned vessels, unmanned vehicles, and rotary-wing UAVs can achieve hovering, while unmanned vehicles such as fixed-wing UAVs must maintain a certain speed. In order to adapt to the latter type of unmanned vehicle, the algorithm must have good real-time performance.

Convergence: After the algorithm is completed, the FCS needs to immediately control the unmanned vehicles in order to form the formation generated using the result. Therefore, the result of the algorithm is required to be a convergence result that meets the indicators.

Distributed Computing: according to the characteristics of the unmanned vehicle formation and the analysis of the first part of this paper, the formation generation algorithm should be distributed.

## 3. Multi-Agent Hybrid Social Cognitive Optimization Based on the Internet of Things (IoT)

After a comprehensive analysis of the formation generation problem described above, we propose an algorithm that combines group intelligence and multi-agent technology: multi-agent hybrid social cognitive optimization (MAHSCO).

### 3.1. Basic Introduction to MAHSCO

The basis of MAHSCO is the social cognitive optimization (SCO) algorithm proposed by Xie [21]. SCO is an advanced swarm intelligence optimization algorithm. Unlike traditional swarm intelligence algorithms such as the ant colony algorithm and the particle swarm optimization algorithm, the SCO algorithm refers to the learning process of members of human society; the ant colony algorithm and the particle swarm algorithm mimic less intelligent insects and flocks, while the SCO algorithm simulates the cognitive process of human society, which has higher intelligence and stronger sociality. Therefore, the SCO algorithm is expected to have stronger intelligence and better complex problem-solving ability.

The basic principle of the SCO algorithm is to set the solution to the problem as a knowledge library and set the members to solve the problem as learning agents. Learning agents constantly seek a better solution to a problem by imitation learning and observation learning. Through continuous cognition and learning, learning agents finally obtain the best solution to the problem. It can be seen that this process is highly similar to the process of a human learning new knowledge.

Early swarm intelligence optimization algorithms [22,23,24] mostly simulated natural phenomena. Compared with these, SCO methods, which simulate human cognitive processes, are likely to have better comprehensive performance. The SCO algorithm has been applied to solve the following problems: system of nonlinear equations, nonlinear complementarity problem, reliability allocation problem, and composition web service selection problem [25]. According to the application of the above problems, SCO has the prominent advantage of high stability. According to [26], although SCO is a centralized algorithm, it has the potential to be distributed.

Considering the social attributes of human cognition allowed the SCO algorithm to be improved by adding chaos learning and elite learning into the basic SCO algorithm [27,28]. In the SCO algorithm, each learning agent occupies the same status. However, in human society, the cognitive abilities of each member are very different, and different members adopt different cognitive and learning strategies to maximize the efficiency of the entire group. Therefore, study [29] added this feature to the traditional SCO algorithm, dividing the learning agents into elite agents, ordinary agents, and low-level agents. The elite agents adopt an elite learning strategy, the ordinary agents adopt an imitation learning and observation learning strategy in the basic SCO algorithm, and the low-level agents adopt a chaotic learning strategy. The improved algorithm is called the hybrid social cognitive optimization (HSCO).

In order to adapt to the characteristics of multiple unmanned vehicle formation and distribute the HSCO algorithm, this paper introduces a multi-agent system into the HSCO algorithm.

Due to the openness and flexibility of the multi-agent system, it is easy to combine with other artificial intelligence algorithms and to demonstrate its structural advantages and interaction capabilities in the new algorithm. Studies [30,31] have given the method of distributed multi-agent system. Therefore, the combination of the HSCO algorithm and a multi-agent system is logical.

The addition of the IoT solves the only problem: distributed computing and communication. Therefore, in this paper, distributed computing and communication are combined to propose the MAHSCO algorithm, and the multiple unmanned vehicles can achieve better collaboration and quick solution to the formation generation problem by exchanging information about their respective knowledge libraries.

### 3.2. Basic Steps of MAHSCO

**Figure 4:**
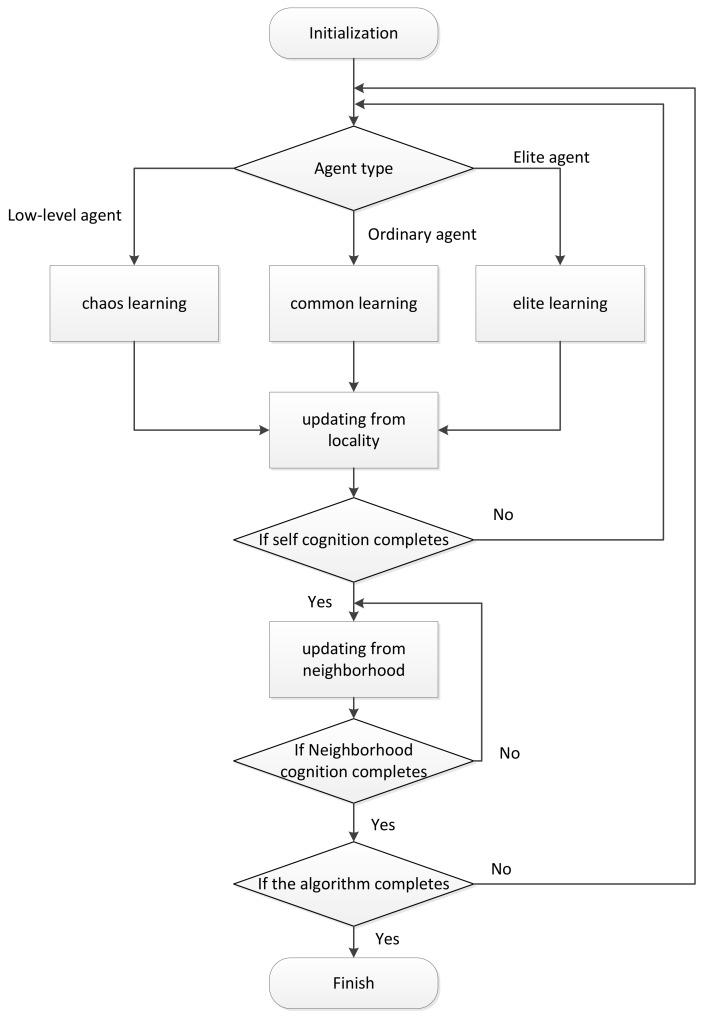
Basic steps of the multi-agent hybrid social cognitive optimization (MAHSCO) algorithm.

The MAHSCO algorithm is generally divided into the following steps: initialization, self-cognition, neighborhood learning, and conclusion making. The general idea of the algorithm is as follows. At the beginning of the algorithm, each agent randomly generates a solution. As the algorithm proceeds, each agent continuously learns to obtain a better solution, and finally produces a relatively optimal solution. The flow chart of MAHSCO is shown in Figure 4. 

#### 3.2.1. Initialization

Before the MAHSCO begins, it is necessary to abstract the problem to be solved into a mathematical optimization problem, that is, to propose an objective function. The objective function varies with the different problems faced. In this paper, by transforming the form of the objective function, all the problems studied are transformed into the problem of solving the maximum value of the objective function. 

After the objective function is determined, each member of the group randomly generates a knowledge library. The knowledge library contains a certain number of solutions that are randomly distributed within the allowable range. The quality of the solution depends on the objective value when it is adopted for the objective function. In this paper, since all the objective functions are transformed to seek the maximum value, the solution which makes the objective function achieve a greater value is a better solution. After that, each member generates a certain number of learning agents. Each learning agent randomly learns one solution in the knowledge library (each agent learns a different solution). The current solution of the learning agent is recorded as SCA.

#### 3.2.2. Self-Cognition

Self-cognition is the process by which learning agents choose better solutions through various learning methods. In this process, the learning agents are divided into three types as described above: elite agents, ordinary agents, and low-level agents. Each agent’s solution produces a value for the objective function. Sort the agents according to the size of the generated value. Usually the top 5% of the agents are defined as elite agents. The last 5% of the agents are defined as low-level agents. The rest are defined as ordinary agents. Different agents use different learning methods:

• Elite agents use elite learning

Let the performance value of the optimal solution in the knowledge library of the current formation member be $GB_{p}$. The performance value of the agent’s current solution is recorded as SCA. SCA should be compared with $GB_{p}$. If $GB_{p}$ is better than SCA, center on $GB_{p}$ and use SCA as a reference point for neighborhood search. The new solution obtained is recorded as $TS_{o}$. If $GB_{p}$ is not better than SCA, make $GB_{p}$ equal to SCA. The neighborhood search method described above is as follows:

Let $x_{1}$ be the reference point and $x_{2}$ be the center point, then the new solution $x^{\prime }$ is generated by the following equation:

(1) $$x^{\prime }=x_{1}+2Rand()(x_{2}-x_{1})$$

• Ordinary agents use common learning

Common learning is an abstraction and simulation of the conventional learning methods of human beings. In the MAHSCO algorithm, it is used as a self-cognition method for ordinary agents.

Humans and some other intelligent creatures have the ability to imitate the same kind, which allows the populations of these organisms to share knowledge quickly. When an agent of the population acquires advanced knowledge, imitation can quickly spread this knowledge and increase the overall knowledge level of the population. This is the principle of imitation learning.

And the more advanced aspect of human wisdom is that humans can predict future behavior by constructing predictive models of behaviors by observing others’ behaviors and their consequences. This makes it unnecessary for humans to try every kind of behavior in person, and to gain new knowledge by thinking about the behaviors that have already been done. This method is called observation learning.

Based on the above analysis, the common learning in MAHSCO is also divided into two parts: imitation learning and observation learning. In imitation learning, the agent selects some existing knowledge points in the knowledge base to imitate, so as to obtain new knowledge. In observational learning, the agent analyzes the results of the imitation learning and the knowledge that it has mastered before, so as to obtain new knowledge. The implementation details of the method are as follows.

Imitation learning: Randomly choose $\tau_{B}$ solutions in the knowledge library ($\tau_{B}$ is the competition width, usually chosen as 2). The solution chosen cannot be the same as the current solution of the learning agent. Then, choose the best among the $\tau_{B}$ solutions, denoted as $TS_{p}$.

Observation learning: Compare the best solution $TS_{p}$ generated in imitation learning with the current solution SCA of the learning agent. Take the better solution in $TS_{p}$ and SCA as the center point and use another point as the reference point for neighborhood search. The specific method of neighborhood search in common learning is consistent with the neighborhood search in elite learning described above. The updated equation is shown in Equation (1). The newly generated point is recorded as $TS_{o}$. If the generated $TS_{o}$ is better than the previous global optimal solution $GB_{p}$, the value of $TS_{o}$ is given to $GB_{p}$.

• Low-level agents use chaos learning

The main purpose of chaos learning of low-level agents is to ensure the randomness and ubiquity of the algorithm. The low-level agent does not observe, and only generates a chaotic solution through the Tent map. The specific method is as follows:

Step 1: randomly generate initial value $x_{0}$, ($0\leq x_{0}\leq 1$);

Step 2: Iterative calculation should be performed by the following equation:

(2) $$x_{k+1}=\left\{\begin{array}{ll} 2x_{k}, & 0\leq x_{k}\leq 1/2\\ 2(1-x_{k}), & 1/2<x_{k}\leq 1 \end{array}\right.$$

The number of iterations is chosen as 300. The iteration result is recorded as $x_{M}$. In the iterative calculation process, if $x_{i}=\left\{0,0.25,0.5,0.75\right\}$ or $x_{i}=x_{i-k},k=\left\{1,2,3,4\right\}$, $x_{i}$ should add a small amount: $x_{i}=x_{i-k}+\varepsilon$. This is to avoid the iterative process falling into small cycles or unstable points [32];

Step 3: Calculate the chaos solution by the following equation:

(3) $$f(x)=V_{\min}+x_{M}\cdot |V_{\max}-V_{\min}|$$

where $V_{\max},V_{\min}$ are the upper and lower bounds of the interval. The newly generated chaos solution is recorded as $TS_{o}$.

#### 3.2.3. Agent’s Solution and Knowledge Library Updating from Locality

If the new solution $TS_{o}$ calculated in Step 2 is better than the current solution of the agent, the agent chooses the new solution $TS_{o}$ as its own solution.

Then select $\tau_{W}$ solutions from the knowledge base ($\tau_{W}$ is usually chosen as 4), remove the worst solution $TS_{w}$, and replace it with the point $TS_{o}$ generated in the previous self cognition.

Since there are multiple learning agents in each member, process 4 can only be performed after each agent has performed processes 2 and 3 once.

#### 3.2.4. Knowledge Library Updating from Neighborhood Members

Select $\tau_{C}$ worst solutions from the knowledge library ($\tau_{C}$ is usually chosen as 4) and replace them with the $\tau_{C}$ solutions generated in the neighborhood member. If there are multiple neighborhood members, repeat the above replacement. The neighborhood members are derived from the current communication topology.

After each member has completed processes 3.2.2, 3.2.3, and 3.2.4, the entire group finishes a complete social cognition.

In theory, MAHSCO must be able to get a convergent solution. In practical applications, due to time constraints, the following two conditions are often used as the end of the algorithm:

(1) The value of the objective function corresponding to the optimal solution obtained by the algorithm reaches the given requirement.
(2) Specify a maximum number of loops, the algorithm ends when the number of algorithm loops reaches this maximum number.

### 3.3. Proof of MAHSCO Convergence

**Definition** **1.** *Suppose there is a non-empty class* ℑ *, and its elements are sets. If the following three conditions are met,* ℑ *is a* σ− *field:*

*(1)*  Ω∈ℑ *;*
*(2)*  *If*$E_{j}\in ℑ,j=1,2,\ldots$*,*$\overset{\infty }{\underset{j=1}{\cup }}E_{j}\in ℑ$;
*(3)*  *If* E∈ℑ *,* $E^{c}\in ℑ$
  *where*Ω*is the universal set, and*$E^{c}$*is the complementary set of*E.

**Definition** **2.** *If* μ *is a set function defined in the non-empty class* l *and meets the following three conditions simultaneously:*

*(1)*  *For any* E∈l *, there is:*
  (4) 0≤μ(E)≤∞
*(2)*  *If* ϕ∈l *,* μ(ϕ)=0 *;*
*(3)*  *If*$E_{j}\in l,j=1,2,\ldots$*,*$\overset{\infty }{\underset{j=1}{\cup }}E_{j}\in l$*when*$E_{k}\cap E_{j}=\phi ,\forall k\neq j$*, then*$\mu \left(\overset{+\infty }{\underset{k=1}{\cup }}E_{k}\right)=\sum_{k=1}^{+\infty }\mu \left(E_{k}\right)$.

The set function μ is called the measure on the non-empty class l. When Equation (4) is replaced by Equation (5), the set function μ is called the probability measure on the non-empty class l.

(5) 0≤μ(E)≤1

Suppose there is an optional set Ω, ℑ is the σ− field consisting of subsets of Ω, μ is the probability measure on ℑ, then the triple $\left(\Omega ,ℑ,\mu \right)$ is called the probability space.

The process of using a random algorithm to give convergence criteria is as follows:

(1) Randomly select the initial point $z_{0}\in S$, let k=0;
(2) Generate a vector $\zeta_{k}$ on the sample space $\left(R^{n},B,\mu_{k}\right)$;
(3) Calculate $z_{k+1}=D\left(z_{k},\zeta_{k}\right)$, choose $\mu_{k+1}$, let k=k+1 and implement iterative calculations, where $\left(R^{n},B,\mu_{k}\right)$ refers to the probability space of the algorithm at the kth generation, B refers to the σ− field of a certain subset of $R^{n}$, $\mu_{k}$ refers to the probability measure on B, and D refers to the Iterative approach of the algorithm.

**Definition** **3.** *Suppose there is a subset of*$R^{n}$*being*M*. If it satisfies the following three conditions, it is called the support set of probability measure*$\mu_{k}$:

*(1)*  $\mu_{k}\left(M_{k}\right)=1$;
*(2)*  *Randomly select the point sequence*${\left\{y_{k}\right\}}_{k=1}^{+\infty }\subseteq M_{k}$*, for any converged subsequence*${\left\{y_{j}^{k}\right\}}_{j=1}^{+\infty }$*, there is*$\underset{j\to +\infty }{\lim}y_{j}^{k}\in M_{k}$;
*(3)*  *If*$N\in R^{n}$*satisfies 1 and 2,*$M_{k}\subseteq N$.

Only by ensuring that the new individuals produced by D are superior to the previous individuals can the effectiveness of the random algorithm be ensured. Therefore, make the following assumptions for random algorithms:

**Assumption** **1.** $f\left(D\left(z,\xi \right)\right)\leq f\left(z\right)$*and if*ξ∈S*, then*$f\left(D\left(z,\xi \right)\right)\leq f\left(\xi \right)$.

*The global convergence of the random algorithm means that the sequence*${\left\{f\left(z_{k}\right)\right\}}_{k=1}^{\infty }$*should converge to*ψ. $\psi =\inf\left\{x|v\left(z\in S|f\left(z\right)<x\right)>0\right\}$.

**Definition** **4.** *Suppose the* ε *in the algorithm makes sense in the following areas:*

(6) $$R_{\varepsilon }=\left\{z\in S|f\left(z\right)<\psi +\varepsilon \right\}$$

In Equation (6), ε>0. If the algorithm finds a point in $R_{\varepsilon }$, the algorithm is considered to have found an acceptable point. The error at this point is ε;

**Assumption** **2.** *Suppose that*S*has any Borel subset*A*, if its measure satisfies the condition*$v\left(A\right)>0$*then:*

(7) $$\prod_{k=0}^{\infty }\left(1-\mu_{k}\left(A\right)\right)=0$$

*where*$\mu_{k}\left(A\right)$*is calculated by the probability measure*$\mu_{k}$.

**Theorem** **1**  **[33].** *Assume that the objective function*f*is measurable,*S*is a measurable subset within the field and satisfies Assumptions 1 and 2. Let the solution sequence of the algorithm iteration be*${\left\{z_{k}\right\}}_{k=1}^{+\infty }$, then:

(8) $$\underset{k\to \infty }{\lim}P[z_{k}\in R_{\varepsilon }]=1$$

*where*$P[z_{k}\in R_{\varepsilon }]$*is the probability of the solution*$z_{k}\in R_{\varepsilon }$*generated by the*kth*step of the algorithm. Theorem 1 gives the necessary and sufficient conditions for the stochastic optimization algorithm to globally converge with probability 1.*

Define the function D as follows:

(9) $$D\left(p_{gk},x_{ik}\right)=\left\{\begin{array}{ll} p_{gk}, & f(g(x_{ik}))\geq f(p_{gk})\\ g(x_{ik}), & f(g(x_{ik}))<f(p_{gk}) \end{array}\right.$$

where $p_{gk}$ refers to the global best of step k, $g(x_{ik})$ refers to the updating of the ith agent, and $x_{ik}$ refers to the location of the agent i at the kth step. Since the MAHSCO algorithm retains the optimal solution in the learning process in the knowledge library, according to the definition of function D, it can be shown that the MAHSCO algorithm satisfies Assumption 1.

For the learning agent group, the establishment of Assumption 2 is equivalent to the algorithm satisfying Equation (10):

(10) $$S\subseteq \overset{N}{\underset{i=1}{\cup }}M_{i,k}$$

where $M_{i,k}$ is the support set of point i of the algorithm at step k.

For the MAHSCO algorithm, in all learning iteration points, some points are updated according to chaos learning. Chaotic sequences have global ergodicity. For the points with chaos learning, $M_{i,k}=S$. Therefore, the MAHSCO algorithm satisfies $S\subseteq \overset{N}{\underset{i=1}{\cup }}M_{i,k}$, and also satisfies Assumption 2.

In summary, the MAHSCO algorithm converges globally to the global optimal solution with a probability of 1.

## 4. Generation of Unmanned Aerial Vehicle (UAV) Formation

In the aforementioned unmanned vehicle formation problem, the MAHSCO algorithm was used to solve the formation generation problem. According to the analysis in Section 2, part C, large formations are all composed of small formations with 6–8 members. Considering the cost of flight verification, the conducted simulation and flight verification took the number of members as six, i.e., the minimum number that could reflect the characteristics of the formation generation problem.

When the MAHSCO algorithm is applied to the UAV formation generation problem, each UAV becomes a “member” as mentioned in Section 3, that is, each UAV generates a knowledge library and several learning agents. The knowledge contains a number of solutions which are the available formations in this problem. The “self-cognition” is carried out in each UAV and the “neighborhood learning” is implemented among the UAV group.

### 4.1. Initial Conditions

**Figure 5:**
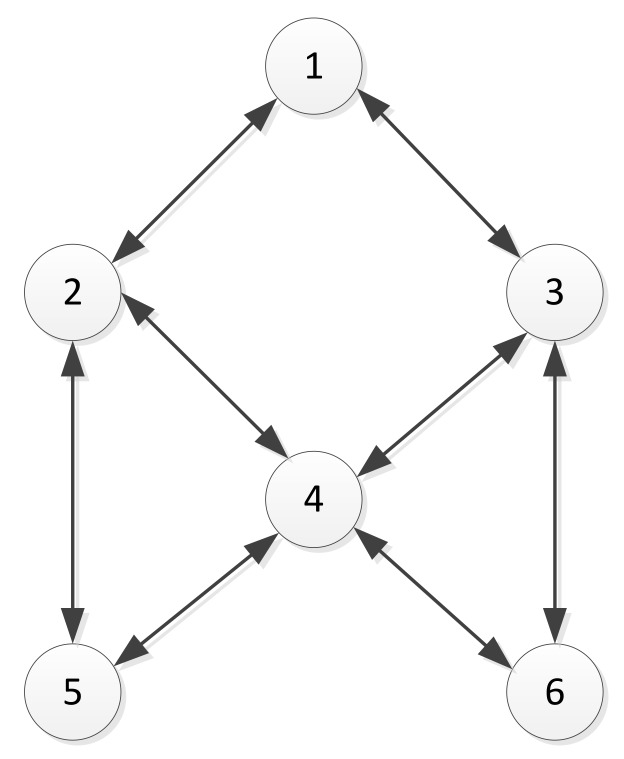
Communication topology of a typical formation with six unmanned aerial vehciles (UAVs). The circles represent the UAVs, the numbers in the circles are the UAV numbers, and the arrows indicate that there is communication between two UAVs.

Before the formation calculation simulation, it is necessary to confirm the initial situation of the six UAVs. In the UAV formation, it is not necessary for every pair of members to communicate with each other; communication is usually only made between neighboring members. The communication topology relationship of each UAV in the MAHSCO algorithm has an effect on the cognitive learning relationship among the UAVs. However, the communication relationship does not affect the implementation method of the algorithm. Therefore, in this simulation, it was assumed that six UAVs are in a common polygon formation. The communication topology is shown in Figure 5.

In Figure 5, the arrows representing communication between pairs of UAVs are all the two-way arrows, since the radios that are currently in common usage can achieve two-way communication. According to the communication topology shown in Figure 5, the communication relationship between the UAVs can be represented by the following matrix C:

(11) $$C=\left[\begin{array}{cccccc} 0 & 1 & 1 & 0 & 0 & 0\\ 1 & 0 & 0 & 1 & 1 & 0\\ 1 & 0 & 0 & 1 & 0 & 1\\ 0 & 1 & 1 & 0 & 1 & 1\\ 0 & 1 & 0 & 1 & 0 & 0\\ 0 & 0 & 1 & 1 & 0 & 0 \end{array}\right]$$

When the matrix element $a_{ij}$ is 1, it means that the i-th UAV and the j-th UAV have communication. Conversely, when $a_{ij}$ is 0, there is no communication between the two UAVs. This matrix is used in the MAHSCO formation generation algorithm described later.

It is worth mentioning that, although the number of unmanned vehicles and the complexity of the communication topology in formation have little impact on the MAHSCO algorithm itself, the application of MASHCO is limited when the formation size is too large and the communication topology is too complex, since the neighborhood cognition step is realized through actual radio communication between different unmanned platforms in practical engineering applications. In fact, as long as it is a connected topology, it can guarantee the normal operation and convergence of the algorithm. The lower the connectivity is, the higher the communication frequency requirements. However, when the topology changes constantly, there is a possibility that the algorithm cannot converge. Algorithmic improvements in topology changes will be used as future research content.

### 4.2. Optimization Indicators

Because the MAHSCO algorithm is used to generate the formation of the UAVs, the evaluation indicators of the formation are the optimization indicators of the MAHSCO algorithm. The main basis for the formation evaluation indicators is the three formation principles described in Section 2 of this paper. Different tasks will have different specific requirements. For example, crossing a canyon requires the UAVs to strictly maintain the column formation, some search tasks require the UAVs to strictly synchronize in the horizontal direction, while sometimes the limitation of communication distance requires the UAVs to be as close to each other as possible. The corresponding formation parameter indicators include the maximum vertical distance of the formation, the maximum horizontal distance of the formation, the maximum area of the 2D formation, the maximum area that the formation can detect, and the range of angle at which the weak members of the formation can be detected. The latter part of this article will simulate the optimization of several typical indicators.

### 4.3. Constraint Condition

For the formation optimization of UAV formation, the most important constraint is the distances between UAVs. According to study [34], the maneuverability in all directions is limited for fixed-wing UAVs. The necessary distance between the UAVs is often called the safety distance. According to the conclusion of study [19], the safety distance of a UAV formation is mainly related to the following factors: sensor and control error, maneuverability, network quality, environment, and mission situation. This paper does not specifically discuss the analysis and maintenance of UAV safety distance, but only sets a specific distance value as a constraint for UAV formation; formations with a minimum member distance less than this value will not be allowed. This value is also the main constraint of the MAHSCO algorithm.

## 5. Simulations and Results

### 5.1. Algorithm Parameter

When using the MAHSCO algorithm to optimize the formation of six UAVs, each UAV member generates a knowledge library containing 350 solutions. Each UAV member chooses 35 agents for cognitive learning. After selecting a certain UAV as the reference point, the 2D plane rectangular coordinate system is adopted, and the position of each of the other UAVs is determined by the distance in the x and y directions. Therefore, one solution group contains 10 solutions. In the algorithm, the ratios of elite agents, ordinary agents, and low-level agents are 5, 90, and 5%, respectively. The competition width $\tau_{B}$ is selected as 2, the local update width $\tau_{W}$ is selected as 4, and the neighbor update width $\tau_{C}$ is selected as 4.

### 5.2. Conditional Parameter

According to the actual flight experience, the safety distance of a certain kind of small UAV could be approximated to 8 m. In this condition, the two UAVs with the largest distance among the six must be kept at a distance of at least 40 m to ensure a solution to the formation problem. After appropriately expanding this range, the solution range for each solution is −100 to 100.

### 5.3. Simulation Result in Each Optimizing Indicator

In order to verify the effectiveness of the algorithm, the following indicators were used:

(1) Minimum formation horizontal distance

**Figure 6:**
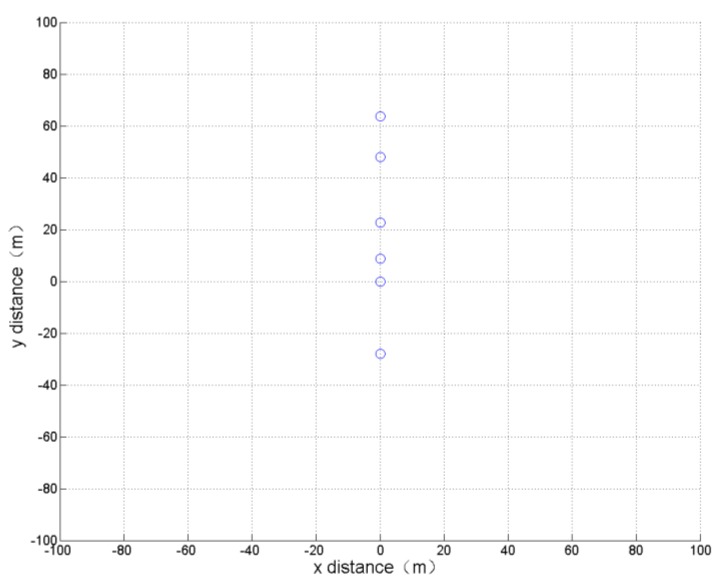
Formation optimization results of minimum formation horizontal distance.

In this simulation, only the formation horizontal distance is optimized in order to obtain the smallest formation horizontal distance without paying attention to other parameters. After 800 cycles of learning calculation, the optimization results and the formation obtained are shown in Table 1 and Figure 6.

It can be seen that the result of the optimization is consistent with the actual experience, resulting in a single vertical formation.

(2) Minimum formation vertical distance

**Figure 7:**
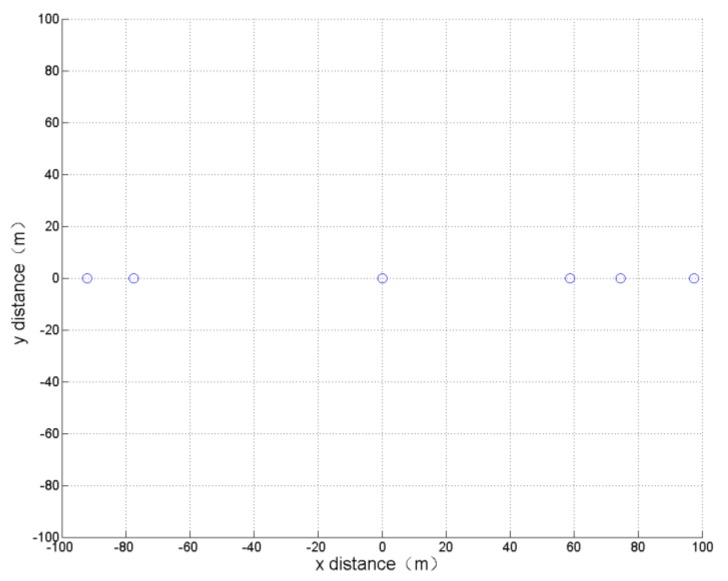
Formation optimization results of minimum formation vertical distance.

In this simulation, only the formation vertical distance is optimized in order to obtain the smallest formation vertical distance without paying attention to other parameters. The optimization results and the formation obtained after 800 cycles of learning calculation are shown in Table 2 and Figure 7.

It can be seen that the result of the optimization is consistent with the actual experience, resulting in a single horizontal formation.

(3) Minimum span

**Figure 8:**
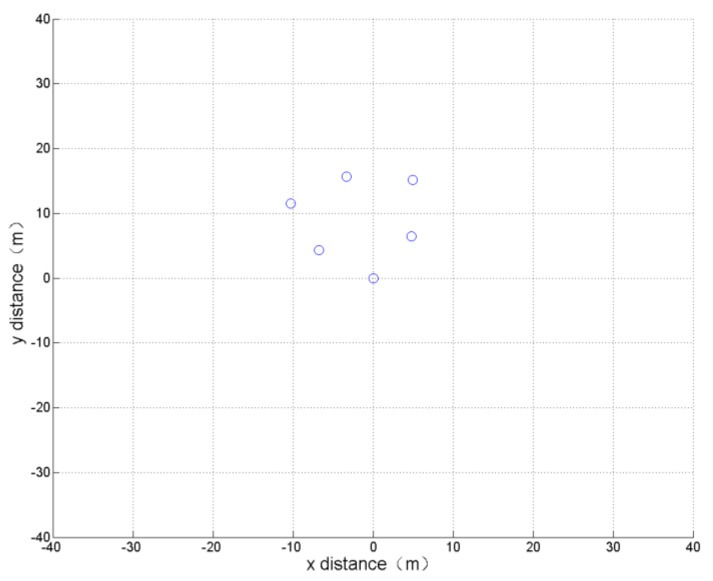
Formation optimization results of minimum span.

The optimization indicator of this simulation is: the span of the UAV formation is the smallest. This means that the two UAVs that are farthest apart in the formation maintain a small distance. The optimization results and the formation obtained after 800 cycles of learning calculation are shown in Table 3 and Figure 8.

The simulation results show that all the UAVs in the generated formation are closely clustered, which is consistent with the actual experience.

(4) Minimum average distance

**Figure 9:**
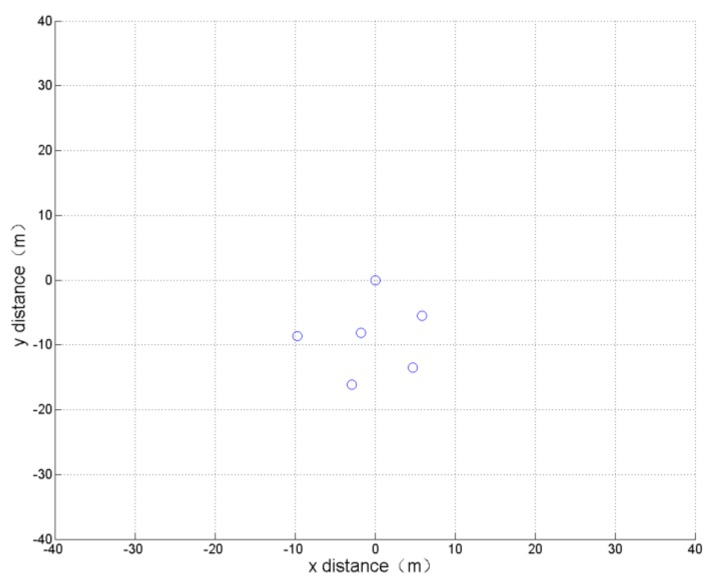
Formation optimization results of minimum average distance.

The optimization indicator of this simulation is: the root mean square (rms) value of the distance between all UAVs and the reference UAV is the smallest. This means that the formation of the UAVs should be relatively tight. The optimization results and the formation obtained after 800 cycles of learning calculation are shown in Table 4 and Figure 9. 

The simulation results show that all the UAVs in the generated formation are closely clustered, which is consistent with the actual experience.

### 5.4. Analysis Results

In the simulation experiment of the above four formations, the formation generated by the MAHSCO algorithm is consistent with the theoretical optimal formation. Moreover, the distance between the unmanned aerial vehicles in the generated formation is less deviated from the theoretical value, and the precision is high.

### 5.5. Comparison of MAHSCO and HSCO

As described above, the main difference between MAHSCO and HSCO is whether the neighborhood recognition is performed or not. The HSCO only needs one unmanned platform to calculate, while MAHSCO needs to calculate on each platform. However, due to the mutual communication between platforms, the computation amount of each platform in the MAHSCO algorithm can be much less than the total computation amount in the HSCO algorithm. The corresponding disadvantage is that MAHSCO needs more cycles to achieve the same precision as HSCO if the amount of computation in each learning cycle is equal. Taking the first optimization in Section 5.3. as an example, the number of cycles required for both algorithms to achieve the same precision result is shown below. (The minimum formation horizontal distance below −1 × 10^−10^ was taken as the end condition of optimization. In order to keep the computation amount of each cycle equal to that of MAHSCO, the agent amount in the HSCO for comparison was taken as 210.)

It can be seen from the data in Table 5 that MAHSCO requires more cycles than HSCO under similar conditions. However, the increase in the number of cycles is not much, considering that the computation amount of each unmanned platform is only 1/6 of the original. Also, since all the calculations in the HSCO are performed in one unmanned platform, the entire formation cannot be implemented once it fails. Therefore, MAHSCO is a better algorithm for the generation of UAV formations.

### 5.6. Robustness and Fault-Tolerant Analysis of MAHSCO

In MAHSCO, the operation of the algorithm relies on the transfer of information between members. In practical applications, information transmission is achieved through radio stations. Therefore, under actual circumstances, MAHSCO will face errors caused by transmission delay, packet loss and bit error.

In engineering applications, we add transmission feedback and verification. When a member needs to send a message, it will continue to send until it receives feedback from all neighboring members or reaches the upper limit of the sending time. In this case, the probability of a message being erroneously transmitted is low, but more information is lost. Although it turns out that the final algorithm will still reach convergence, it will reduce the optimization efficiency of MAHSCO. In order to verify the operational efficiency of MAHSCO in the case of partial information loss, we added 5%, 10% and 20% of the packet loss in the algorithm information transmission. Still taking the first optimization in Section 5.3 and the same condition in Section 5.5 as an example, the comparison that the number of cycles required achieving the same operational precision of no packet loss, 5% packets loss, 10% packets loss and 20% packets loss are shown in Table 6.

It can be seen from the data in Table 6 that MAHSCO’s efficiency has dropped when information is lost. However, even if the packet loss rate reaches 20%, it can converge faster to the specified accuracy. And, by comparing with Section 5.3, the distributed decision is proved still better than the centralized decision. (The total number of cycles is less than 2 times, but the calculation amount of each member is 1/6 of the original.) The main reason is that MAHSCO retains the characteristics of member self-learning.

## 6. Flight Verification and Result

**Figure 10:**
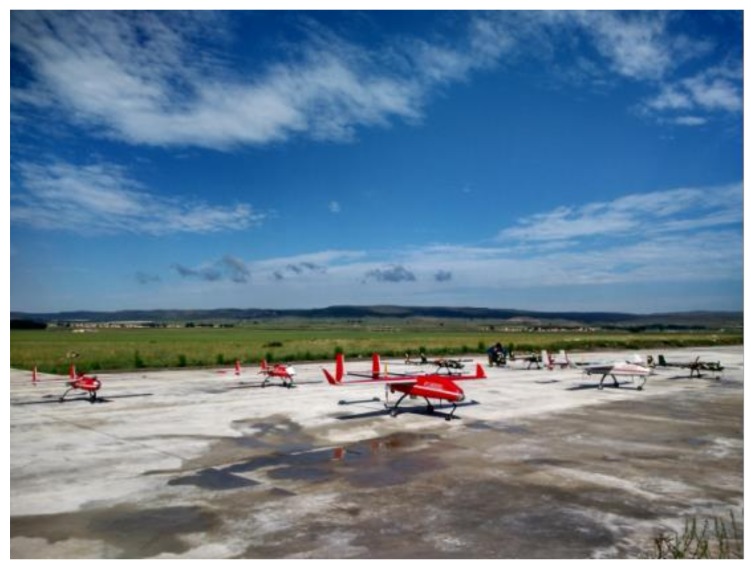
UAVs used for flight verification.

**Figure 11:**
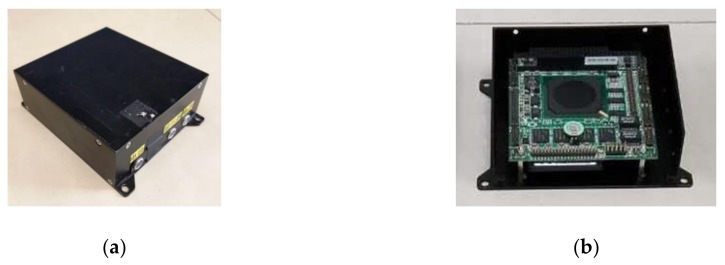
Formation controller based on small embedded computer: (**a**) external appearance of the formation controller; (**b**) internal structure of the formation controller.

On the basis of the simulation experiment, an actual formation generation experiment was carried out with small UAVs. Six small UAVs (shown in Figure 10 along with an additional UAV) carrying formation controllers (based on small embedded computers, as shown in Figure 11) and network communication stations respectively carried out the formation flight in the four formations in the simulation. The flight height is about 250 m.

The formation generation software of the algorithm is written in standard C language. Therefore, the program can be easily ported to lightweight applications and devices with scalability and interoperability. The network communication stations used in flight verification adopt time division multiple access (TDMA) technology and broadcast mode. That is to say, when each member sends a message, other members within a certain range can receive it. 

**Figure 12:**
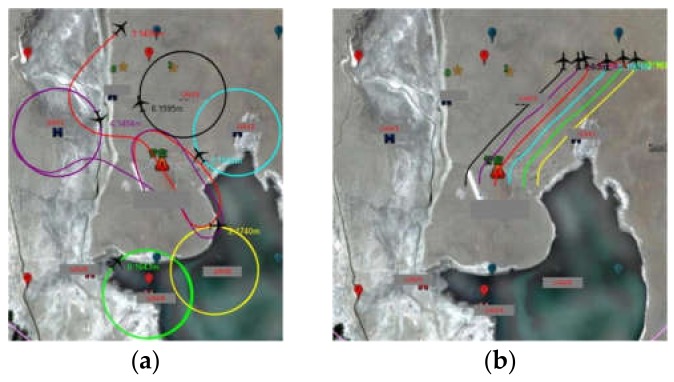

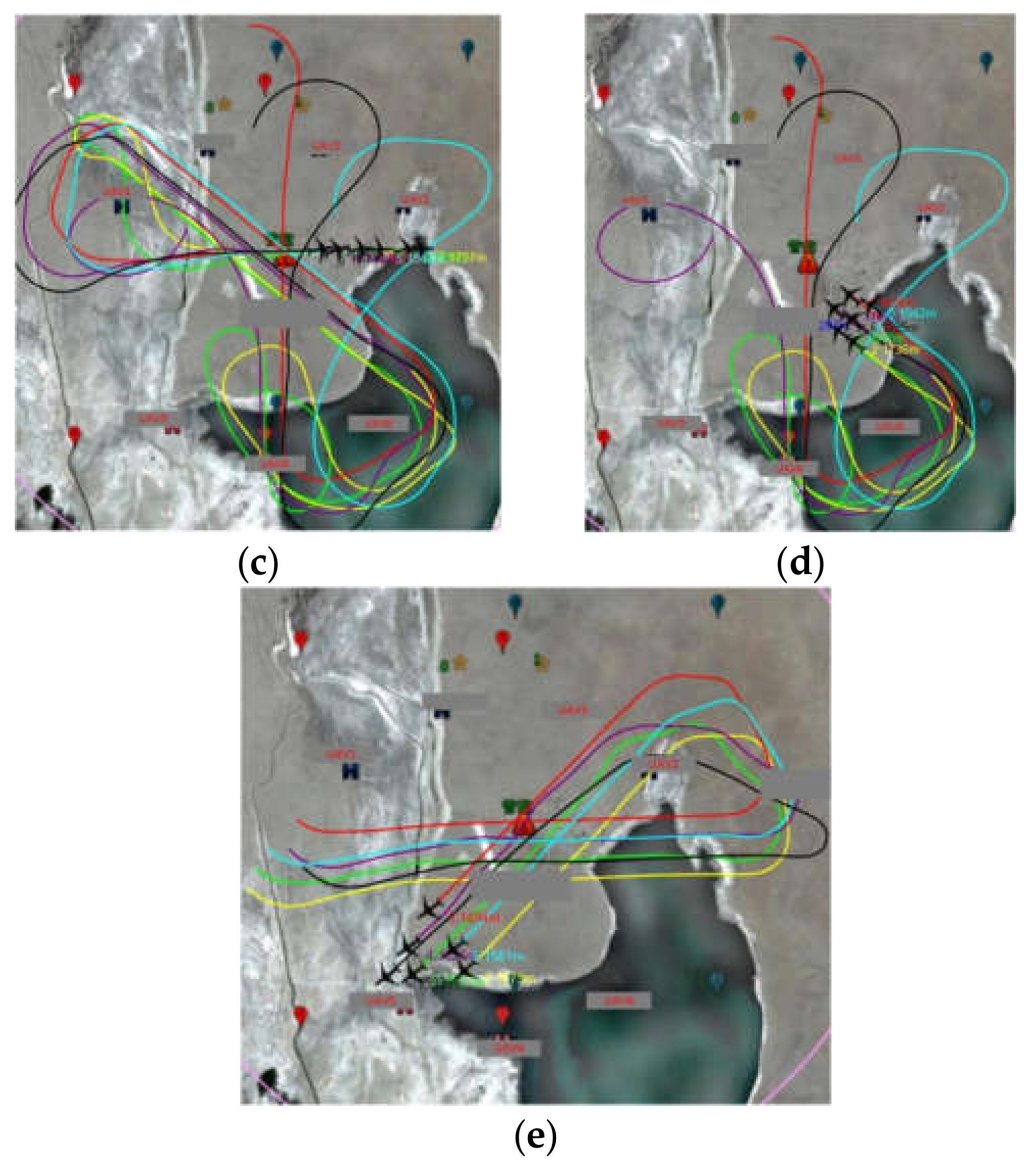
Four formations in the flight verification: (**a**) preparedness of UAVs after take-off; (**b**) formation of minimum formation vertical distance; (**c**) formation of minimum formation horizontal distance; (**d**) formation of minimum average distance; (**e**) formation of minimum span.

When each member has sent a message, a communication cycle is completed. It should be noted that this mechanism requires clock synchronization between stations at a regular interval. In our experiments, the synchronization interval is no more than 1 min. For secure management, the passwords and the secret keys are assigned to the network communication stations offline. When the network is used, the passwords are used for online identity authentication, and the secret keys are used to encrypt the transmission information. Due to difficulties with aerial photography, the ground formation control station was used to observe the formation flight of the UAVs in the air. The formation and actual flight process of the four formations are shown in Figure 12. All formations were generated from the formation controllers on each UAV using the MAHSCO algorithm. Due to the high operating efficiency of the embedded system, the generation time for each new formation was within 15 s, which meets the requirements of small UAV formations. The formation transformation method of the UAVs in the experiment comes from study [35], and the communication control method between UAVs comes from study [36].

## 7. Conclusions

In this paper, a multi-agent hybrid social cognitive optimization (MAHSCO) algorithm based on the IoT is suggested to solve the multi-UAV formation generation problem. Formation principles, UAV formation safety distance, and formation evaluation indicators are considered. The MAHSCO algorithm based on the IoT is proved to be convergent. Computer simulation verification and UAV formation flight verification prove that the MAHSCO algorithm can quickly and accurately generate formations that meet the mission requirements.

In future work, the algorithms could be further optimized to improve speed. Additionally, a formation experiment with a larger scale could be carried out to further extend the application of the algorithm. In order to facilitate engineering applications, we adopted basic identity and authentication method. When the MAHSCO is applied to large-scale complex unmanned systems, advanced dynamic secure management will also be a task for future work.

## Figures and Tables

**Table 1:** Optimization results of indicator 1.

UAV Number	X Distance to UAV 1	Y Distance to UAV 1
1	0	0
2	8.98 × 10^−18^	48.04
3	7.30 × 10^−18^	63.60
4	2.32 × 10^−18^	−27.89
5	1.05 × 10^−17^	8.80
6	1.10 × 10^−17^	22.62

**Table 2:** Optimization results of indicator 2.

UAV Number	X Distance to UAV 1	Y Distance to UAV 1
1	0	0
2	−77.62	2.66 × 10^−22^
3	58.76	−3.75 × 10^−21^
4	97.35	−8.73 × 10^−20^
5	74.55	−3.63 × 10^−20^
6	−92.09	−1.96 × 10^−19^

**Table 3:** Optimization results of indicator 3.

UAV Number	X Distance to UAV 1	Y Distance to UAV 1
1	0	0
2	4.83	6.39
3	4.99	15.17
4	−3.32	15.61
5	−6.74	4.32
6	−10.28	11.50

**Table 4:** Optimization results of indicator 4.

UAV Number	X Distance to UAV 1	Y Distance to UAV 1
1	0	0
2	5.83	−5.52
3	−1.73	−8.15
4	4.70	−13.47
5	−2.92	−16.13
6	−9.71	−8.62

**Table 5:** Comparison of cycles required for the multi-agent hybrid social cognitive optimization (MAHSCO) and hybrid social cognitive optimization (HSCO) algorithms.

Test Number	Cycles Required for MAHSCO	Cycles Required for HSCO
1	498	131
2	397	201
3	363	146
4	528	290
5	396	152
6	392	380
7	413	669
8	473	164
9	446	163
10	498	568
average	410.4	286.4

**Table 6:** Number of cycles required for multi-agent hybrid social cognitive optimization (MAHSCO) to achieve the same accuracy under different packet loss conditions.

Test Number	Cycles Required with no Packet Loss	Cycles Required with 5% Packet Loss	Cycles Required with 10% Packet Loss	Cycles Required with 20% Packet Loss
1	498	869	361	519
2	397	479	531	462
3	363	532	449	513
4	528	313	642	850
5	396	473	466	378
6	392	469	604	387
7	413	454	571	399
8	473	529	434	994
9	446	351	412	454
10	498	433	657	511
average	410.4	490.2	512.7	546.7

## References

[1] Lucas A., Ronnquist R., Howden N., Corke P. Teamed UAVs-a new approach with intelligent agents. Proceedings of the 2nd AIAA “Unmanned Unlimited” Conference and Workshop & Exhibit.

[2] Madhava K., Henry H., Subbarao H., Llinas J. Parametric control of multiple unmanned air vehicles over an unknown hostile territory. Proceedings of the International Conference on Integration of Knowledge Intensive Multi-Agent Systems.

[3] Seiler P., Pant A., Hedrick K. Analysis of bird formations. Proceedings of the 41st IEEE Conference on Decision and Control.

[4] Lei M., Zhou S., Yang X., Yin G.Y. (2012). Complex Formation Control of Large-Scale Intelligent Autonomous Vehicles. Math. Probl. Eng..

[5] Gurfil P., Kivelevitch E. (2007). Flock properties effect on task assignment and formation flying of cooperating unmanned aerial vehicles. Proc. Inst. Mech. Eng. Part G-J. Aerosp. Eng..

[6] Dehghani M.A., Menhaj M.B. (2016). Communication free leader–follower formation control of unmanned aircraft systems. Robot. Auton. Syst..

[7] Zhang T.J. (2017). Unmanned aerial vehicle formation inspired by bird flocking and foraging behavior. Int. J. Autom. Comput..

[8] Kownacki C., Ambroziak L. (2017). Local and asymmetrical potential field approach to leader tracking problem in rigid formations of fixed-wing UAVs. Aerosp. Sci. Technol..

[9] Lee D., Kim S., Suk J. (2018). Formation flight of unmanned aerial vehicles using track guidance. Aerosp. Sci. Technol..

[10] Angelis E.L., Giulietti F., Rossetti G. (2018). Multirotor aircraft formation flight control with collision avoidance capability. Aerosp. Sci. Technol..

[11] Yu W., Chen W., Jiang Z. (2019). Analytical entry guidance for coordinated flight with multiple no-fly-zone constraints. Aerosp. Sci. Technol..

[12] Park C., Cho N., Lee K., Kim Y. (2015). Formation Flight of Multiple UAVs via Onboard Sensor Information Sharing. Sensors.

[13] Chen H.X., Nan Y., Yang Y. (2019). Multi-UAV Reconnaissance Task Assignment for Heterogeneous Targets Based on Modified Symbiotic Organisms Search Algorithm. Sensors.

[14] Sabino S., Horta N., Grilo A. (2018). Centralized Unmanned Aerial Vehicle Mesh Network Placement Scheme: A Multi-Objective Evolutionary Algorithm Approach. Sensors.

[15] Motlagh N.H., Bagaa M., Taleb T. (2017). UAV-based IoT platform: A crowd surveillance use case. IEEE Commun. Mag..

[16] Motlagh N.H., Bagaa M., Taleb T. Uav selection for a UAV-based integrative IoT platform. Proceedings of the IEEE Global Communications Conference.

[17] Yang Q., Yoo S.J. (2018). Optimal UAV path planning: Sensing data acquisition over IoT sensor networks using multi-objective bio-inspired algorithms. IEEE Access.

[18] Zhang Q., Jiang M., Feng Z. (2019). IoT Enabled UAV: Network Architecture and Routing Algorithm. IEEE Internet Things J..

[19] Wu S. (2015). Cooperative Guidance & Control of Missiles Autonomous Formation.

[20] Wu S. (2018). Cooperative Flight Control System.

[21] Xie X., Zhang W., Yang Z. (2002). Social cognitive optimization for nonlinear programming problems. Proc. Mach. Learn. Cybern..

[22] Kirkpatrick S. (1984). Optimization by simulated annealing: Quantitative studies. J. Stat. Phys..

[23] Glover F. (1986). Future paths for integer programming and links to artificial intelligence. Comput. Oper. Res..

[24] Dorigo M., Blum C. (2005). Ant colony optimization theory: A survey. Theor. Comput. Sci..

[25] Hwang S.Y., Lim E.P., Lee C.H. (2008). Dynamic web service selection for reliable web service composition. IEEE Trans. Serv. Comput..

[26] Tang X. (2014). Cross Combination of Swarm Intelligence and Multi-Agent System: Theory, Method and Application.

[27] Bandura A. (2001). Social cognitive theory: An agentic perspective. Ann. Rev. Psychol..

[28] Sun J., Wang S., Chen H. (2014). A guaranteed global convergence social cognitive optimizer. Math. Probl. Eng..

[29] Sun J., Wang S. (2017). Swarm Intelligence Algorithm and Its Application.

[30] Gulati R. (1999). Network location and learning: The influence of network resources and firm capabilities on alliance formation. Strateg. Manag. J..

[31] Sun F., Turkoglu K. (2017). Distributed real-time non-linear receding horizon control methodology for multi-agent consensus problems. Aerosp. Sci. Technol..

[32] Rong H. Study of adaptive chaos embedded particle swarm optimization algorithm based on Skew Tent map. Proceedings of the 2010 International Conference on Intelligent Control and Information Processing.

[33] Solis F.J., Wets R.J.B. (1981). Minimization by random search techniques. Math. Oper. Res..

[34] Wu S. (2013). Flight Control System.

[35] Wen Y., Wu S., Liu W. (2017). A Collision Forecast and Coordination Algorithm in Configuration Control of Missile Autonomous Formation. IEEE Access.

[36] Cai D., Wu S., Deng J. (2017). Distributed Global Connectivity Maintenance and Control of Multi-Robot Networks. IEEE Access.

